# Hepatic benefits of sodium-glucose cotransporter 2 inhibitors in liver disorders

**DOI:** 10.17179/excli2023-6022

**Published:** 2023-04-21

**Authors:** Habib Yaribeygi, Mina Maleki, Tannaz Jamialahmadi, Seyed Adel Moallem, Amirhossein Sahebkar

**Affiliations:** 1 Research Center of Physiology, Semnan University of Medical Sciences, Semnan, Iran; 2 Urology and Nephrology Research Center, Shahid Beheshti University of Medical Sciences, Tehran, Iran; 3 Surgical Oncology Research Center, Mashhad University of Medical Sciences, Mashhad, Iran; 4 Applied Biomedical Research Center, Mashhad University of Medical Sciences, Mashhad, Iran; 5 Department of Pharmacology and Toxicology, College of Pharmacy, Al-Zahraa University for Women, Karbala, Iraq; 6 Department of Pharmacodynamics and Toxicology, School of Pharmacy, Mashhad University of Medical Sciences, Mashhad, Iran; 7 Biotechnology Research Center, Pharmaceutical Technology Institute, Mashhad University of Medical Sciences, Mashhad, Iran; 8 School of Medicine, The University of Western Australia, Perth, Australia; 9 Department of Biotechnology, Mashhad University of Medical Sciences, Mashhad, Iran

**Keywords:** diabetes mellitus, sodium-glucose cotransporter-2 inhibitors, liver, non-alcoholic fatty liver disorders, fibrosis, hepatocellular carcinoma, cirrhosis

## Abstract

Diabetic patients are at higher risk of liver dysfunction compared with the normal population. Thus, using hypoglycemic agents to improve liver efficiency is important in these patients. Sodium-glucose cotransporters-2 inhibitors (SGLT2i) are newly developed antidiabetic drugs with potent glucose-lowering effects. However, recent limited evidence suggests that they have extra-glycemic benefits and may be able to exert protective effects on the liver. Hence, these drugs could serve as promising pharmacological agents with multiple benefits against different hepatic disorders. In this review, the current knowledge about the possible effects of SGLT2 inhibitors on different forms of liver complications and possible underlying mechanisms are discussed.

## Introduction

The global prevalence of diabetes mellitus (DM) is growing rapidly (Mobasseri et al., 2020[59]). This chronic disorder has adverse effects on most metabolic processes and induces many pathophysiological pathways mediating diabetic complications (Forbes and Cooper, 2013[28]). DM is recognized as a key risk factor for several life-threatening disorders such as cardiovascular and hepatic disorders (Fujii et al., 2020[30]). DM-induced hepatic disorders are a group of complications such as non-alcoholic fatty liver (NAFLD), cirrhosis and fibrosis, hepatocellular carcinoma, acute liver failure, hepatitis, bile duct disease, and jaundice that occur in many patients with uncontrolled DM. These disorders reduce or destroy liver sufficiency and significantly threaten patients' survival or quality of life (Tolman et al., 2007[87]). Thus, hepatic disorders are an important cause of death in patients with DM (Tolman et al., 2007[87]). The value of cirrhosis SMR (standardized mortality ratio; the relative rate of an event compared with the background one) was estimated to be 2.52 compared with 1.34 for cardiovascular complications in diabetic patients (Tolman et al., 2007[87]). Moreover, cirrhosis is suggested as the fourth leading cause of death in diabetic patients (Ramachandran et al., 2017[69]). There are strong relationships between DM and hepatitis as well as hepatocellular carcinoma (Hammerstad et al., 2015[35]; Tingting et al., 2018[86]). Therefore, the management of liver function and prevention of hepatotoxicity in the diabetic milieu is of major importance for the prevention of diabetes-induced hepatic disorders (Tingting et al., 2018[86]).

**Figure 1:**
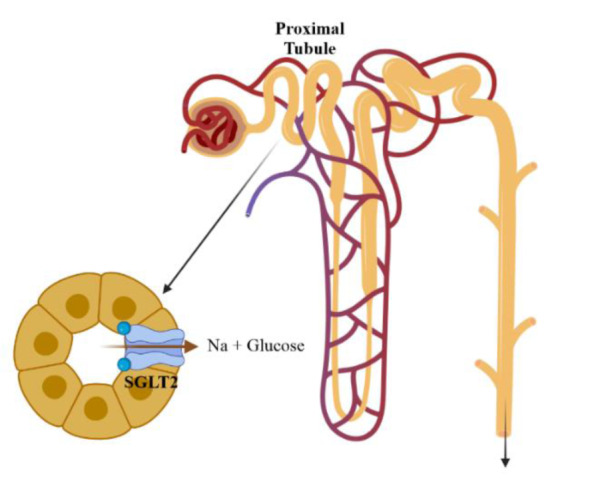
Schematic picture of SGLT2 activity. SGLT2 inhibitors inhibit this process and induce glucose and sodium excretion in urine.

Sodium-glucose cotransporter 2 inhibitors (SGLT2is) are a potent class of antidiabetic drugs that act by glycosuria induction *via* glucose reabsorption inhibition in renal proximal tubules (Figure 1(Fig. 1)) (Yaribeygi et al., 2020[104]). These drugs have a significant impact on blood glucose levels as well as insulin sensitivity in peripheral tissues (Yaribeygi et al., 2020[104]). Moreover, they are now recognized as efficacious agents in diabetic patients with cardiovascular and renal complications owing to their many pleiotropic effects (Yaribeygi et al., 2019[96][98][105], 2020[95][97], 2023[102][101]; Ravindran and Munusamy, 2022[70]). Moreover, recent evidence suggests that SGLT2 inhibitors interact with hepatic functions in the diabetic milieu (Scheen, 2019[76]; Wei et al., 2021[90]). These studies suggest that SGLT2 inhibition modulates liver function through as yet not-well-defined pathways (Wei et al., 2021[90]). In the current study, we discuss the possible liver benefits of SGLT2 inhibitors and elaborate on possible mechanisms involved in such hepatoprotective effects. 

## Classification of Diabetes Mellitus

DM is classically categorized into three main classes: type 1, type 2, and gestational diabetes (American Diabetes Association, 2017[4]). Type 1 DM (T1DM) or insulin-dependent diabetes mellitus (IDDM) mainly refers to reduced circulating insulin due to pancreatic beta cell failure (American Diabetes Association, 2017[4]). Type 2 DM (T2DM) or non-insulin-dependent diabetes mellitus (NIDDM); that is, the most prevalent form of DM; is primarily related to reduced insulin sensitivity and increased insulin resistance in peripheral tissues (American Diabetes Association, 2017[4]). Gestational diabetes is the other form of DM, which develops in pregnant women probably due to hormonal changes (Maraschin, 2012[57]). There are other forms of DM with minor frequency such as LADA (Latent Autoimmune Diabetes in Adults), Maturity-Onset Diabetes of the Young (MODY), secondary diabetes to different conditions such as pancreatitis, and secondary diabetes to some drugs, *e.g.* corticosteroids (American Diabetes Association, 2014[5]; O'Neal et al., 2016[62]). 

## SGLT2 Inhibitors

SGLT2 inhibitors are a newly introduced class of antidiabetic drugs that diminish serum glucose by preventing tubular glucose reabsorption and inducing glycosuria (Davidson and Kuritzky, 2014[23]; Yaribeygi et al., 2018[99]). SGLT2s have two types of active cotransporters (types 1 and 2) that are mainly located in S2 and S3 segments of renal proximal tubules (as well as in small intestines) and reabsorb the filtrated tubular glucose (Figure 1(Fig. 1)) (Yaribeygi et al., 2019[96]). SGLTs can transport glucose, galactose, and sodium ions against concentration gradients (one sodium ion with one D-glucose) (Chao, 2014[15]). Moreover, they are involved in gluconeogenesis, improvement of peripheral insulin sensitivity, glucagon release, and insulin secretion (Han et al., 2008[37]; Ferrannini et al., 2014[27]; Wilding et al., 2014[92]; Kern et al., 2016[45]). Since discovering phlorizin as the first SGLT2 inhibitor, several forms of these agents have been introduced, which all reduce blood glucose to near physiological levels (Chao and Henry, 2010[16]; Clar et al., 2012[20]). SGLT2 inhibitors work completely independently of insulin, and thus have a lower risk of inducing hypoglycemia (Chao, 2014[15]). However, using these drugs may be followed by some side effects such as dehydration, dizziness, hypotension, urinary tract infections, and fainting (Reddy and Inzucchi, 2016[71]). Empagliflozin, dapagliflozin, and canagliflozin are the main approved forms of SGLT2 inhibitors (Reddy and Inzucchi, 2016[71]).

## Physiology of the Liver

The liver is a highly specialized and vital organ that is only developed in vertebrates (Parker and Picut, 2005[66]). It is located in the right upper quadrant of the abdominal cavity, beneath the diaphragm, and on top of the right kidney, stomach, and intestines (Abdel-Misih and Bloomston, 2010[1]; Zong and Friedman, 2014[109]). Anatomically, the liver consists of two greater main lobes each made up of eight segments consisting of about 1000 smaller segments as lobules (Guyton and Hall, 2006[34]). The lobules are connected with the narrower ducts and then to a larger one to form the common hepatic (biliary) duct that transports the produced bile to the gallbladder and duodenum (Guyton and Hall, 2006[34]). It has two distinct blood circulation sources: oxygenated blood flows from the hepatic artery and nutrient-rich blood flows from the hepatic portal vein (Guyton and Hall, 2006[34]). The liver has several important physiological functions in both metabolic and immune-related processes as well as in detoxification, synthesizing, and storing activities (Parker and Picut, 2005[66]; Abdel-Misih and Bloomston, 2010[1]). It is involved in protein and hormone synthesis, glycogen synthesis and storage, blood clotting, vitamin storage, detoxification of the portal and hepatic circulation, clearing drugs and pharmacological agents, and carbohydrate and lipid metabolism (Guyton and Hall, 2006[34]; Zong and Friedman, 2014[109]). Moreover, the liver is involved in cholesterol and lipid homeostasis and the excretion of toxins and ingested heavy metals by producing bile and releasing it into the gallbladder (Guyton and Hall, 2006[34]). Moreover, mesenchymal cells of the liver (known as hepatocytes) control many biochemical activities such as the synthesis and breakdown of small and complex molecules *via* a system of highly fenestrated vessels (Schulze et al., 2019[77]). Estimates have counted about five hundred different activities for the liver (Sakka, 2007[74]). Therefore, a healthy functioning liver is an important issue in body homeostasis.

## Pathophysiology of DM-Dependent Hepatic Disorders at a Glance

**Figure 2:**
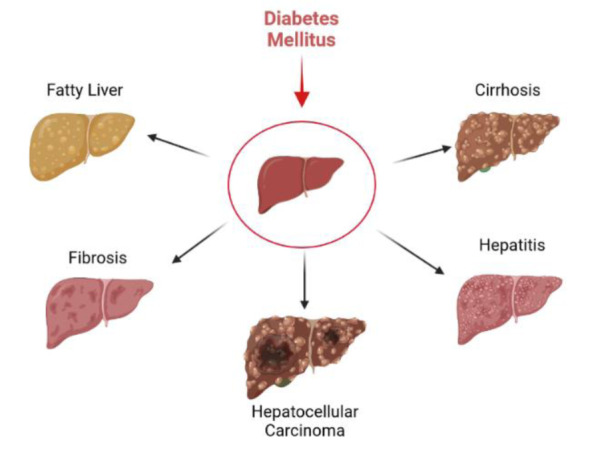
Uncontrolled diabetes induces a variety of hepatic complications.

DM is a strong risk factor for liver function with negative effects on hepatic sufficiency (Hammerstad et al., 2015[35]; Ramachandran et al., 2017[69]; Weiler et al., 2020[91]). DM is considered the main common cause of liver disorders in most epidemiological studies (de Vries et al., 2020;[24] Islam et al., 2020[41]). A wide spectrum of liver complications including abnormal liver enzymes, NAFLD, cirrhosis, non-alcoholic steatohepatitis (NASH), hepatocellular carcinoma, acute liver failure, and icterus are directly related to DM, and are more prevalent in diabetic patients compared to non-diabetic population (Figure 2(Fig. 2)) (Tolman et al., 2007[87]; Leite et al., 2009[49]; Ortiz-Lopez et al., 2012[64]). The exact underlying mechanism inducing liver disorders in the diabetic milieu is not well understood, but the roles of insulin resistance, oxidative stress, and inflammation are more emphasized (Forbes and Cooper, 2013[28]; Mohamed et al., 2016[60]). Since the liver is among the most susceptible organs to DM-dependent oxidative stress, insulin resistance, and subsequent compensatory hyperinsulinemia are the major underlying factors inducing hepatic failure (Bugianesi et al., 2005[14], Manna et al., 2010[56]; Palsamy et al., 2010[65]). Insulin resistance and uncontrolled DM induce pathophysiological pathways inducing free radicals' hyper-production and antioxidant defense system (ADS) weakening, which in turn induce oxidative stress and various injuries to biomolecules such as proteins (including liver enzymes), lipids, and carbohydrates in the liver tissue (Mohamed et al., 2016[60]). 

Moreover, DM induces different inflammatory pathways such as TNF-α (tumor necrosis factor-alpha), c-Jun N-terminal kinase (JNK) and nuclear factor-kappa B (NF-κb) cascades (Han et al., 2006[36]; Banerjee and Saxena, 2012[11]; Mohamed et al., 2016[60]). DM modulates the expression and activity of liver enzymes (Mohamed et al., 2016[60]). For example, cytochrome P450 2E1 (CYP2E1), a main metabolic enzyme mostly expressed in the liver; has higher expression and activity in diabetic and obese individuals and induces excessive metabolism of polyunsaturated fatty acids, which in turn increases cytotoxic byproducts, mitochondrial reactive oxygen species (ROS) and oxidative stress (Leung and Nieto, 2013[51]). Furthermore, DM can cause fat aggregation in hepatocytes and induces NAFLD, which in turn exacerbates liver functions (Saponaro et al., 2015[75]). NAFLD may induce NASH by triggering inflammatory events, and necrotic processes in hepatocytes, and then, prolonged NASH may predispose to cirrhosis and hepatocellular carcinoma at advanced levels (Mohamed et al., 2016[60]). Therefore, proper management of DM could effectively prevent the activation of these pathophysiological pathways and improve liver sufficiency in the diabetic milieu. 

## SGLT2 Inhibitors and Hepatic Disorders

Using SGLT2 inhibitors in patients with diabetes is increasing and they are now recognized as potent hypoglycemic drugs with minor side effects (Choi et al., 2022[19]). Although they have shown some liver benefits in studies (Latva-Rasku et al., 2019[47], Scheen, 2019[76], Hsiang and Wong, 2020[39]), the involved molecular mechanisms are not well understood. In the following sections, we survey for possible liver benefits of SGLT2 inhibitors in different liver disorders and conclude about possible involved mechanisms. 

### Level of liver enzymes 

The levels of liver enzymes including ALT (alanine transaminase), AST (aspartate transaminase), ALP (alkaline phosphatase), and GGT (gamma-glutamyltransferase), along with other conventional liver function tests (LFTs) such as prothrombin time (PT), activated Partial Thromboplastin Time (aPTT), albumin, and bilirubin (direct and indirect) are routine indicators of liver function and health, and are extensively used in the clinic to evaluate the liver health (Gowda et al., 2009[33]). These LFTs indicate different activities of the liver such as transamination, dephosphorylation, protein synthesis, transfer (of different molecules, e.g. gamma-glutamyl functional groups) during cellular pathways, and synthesis of factors involved in hemostasis (Thapa and Walia, 2007[85]; Gowda et al., 2009[33]). LFTs usually change rapidly in response to any stress and pathogen and hence are beneficial screening tools to detect hepatic dysfunctions (Thapa and Walia, 2007[85]; Gowda et al., 2009[33]). 

Limited but more recent evidence has demonstrated that SGLT2 inhibitors can modify the levels of LFTs (Katsiki et al., 2019[44]; Coelho et al., 2021[21]; Simental-Mendia et al., 2021[82]; Zhang et al., 2021[107]). Inoue and coworkers demonstrated that canagliflozin (100 mg/day) significantly improved LFT levels (AST, ALT, and GGT) in T2DM patients with NAFLD (Inoue et al., 2019[40]). Other evidence provided by Seko and colleagues also showed that SGLT2i therapy with canagliflozin (100 mg/day) significantly decreased and normalized the ALT, AST, and GGT levels in serum samples of T2DM patients with NASH (Seko et al., 2018[78]). In a large population of patients with T2DM, canagliflozin therapy was linked to a reduction in serum ALT levels (Bajaj et al., 2018[9]). Moreover, an analysis of pooled data from six clinical trials including 3801 patients with T2DM demonstrated that SGLT2 inhibition by canagliflozin for 26 weeks improved serum LFTs compared with either placebo or sitagliptin therapy in these patients (Leiter et al., 2016[50]). Another meta-analysis of clinical trials (n= 6745) revealed that canagliflozin reduced the levels of ALT, AST, and GGT in the serum of patients with T2DM after 26 or 52 weeks (Li et al., 2018[52]). A more recent meta-analysis including 1950 T2DM patients with or without NAFLD showed that SGLT2i therapy for at least 8 weeks significantly reduced the serum concentrations of AST, ALT, and GGT enzymes (Coelho et al., 2021[21]). Hence, it seems that SGLT2 inhibitors have favorable effects on LFTs (as the main markers of liver health) and can counterbalance their abnormal levels. These changes were mostly accompanied by reduced levels of HbA1c and body weight (Katsiki et al., 2019[44]).

### Liver cirrhosis 

Liver cirrhosis is a life-threatening liver disease in which hepatic tissues are replaced with a scar with permanent damage to the liver (Tsochatzis et al., 2014[88]). It is caused by different pathophysiological mechanisms leading to progressive necrotic and fibrotic processes that induce parenchymal extinction, structurally abnormal nodules, the collapse of hepatic tissues, and distortion of the hepatic vascular network, which in turn enhances resistance to portal blood flow and induces portal hypertension (Pinzani et al., 2011[67]; Tsochatzis et al., 2014[88]). Liver cirrhosis is considered generally an irreversible injury with poor clinical outcomes, but it is preventable and may be ameliorated (Sohrabpour et al., 2012[83]). This disease is closely associated with DM and its risk is increased in the diabetic milieu (Elkrief et al., 2016[26]), but effective diabetes management can decline its risk or delay its occurrence or may reduce its severity (Garcia-Compean et al., 2009[32]; Elkrief et al., 2016[26]). 

There is limited direct evidence assessing the SGLT2i benefits on cirrhosis (Saffo et al., 2020[72]). Saffo and colleagues demonstrated that patients with cirrhosis using SGLT2 inhibitors had improved liver function after at least 761 days of treatment (Saffo et al., 2020[72]). They showed that SGLT2 inhibition reduced portal hypertension and improved liver and renal hypoxia in these patients (Saffo et al., 2020[72]). In another study, SGLT2i therapy was more effective in increasing survival (adjusted hazard ratio 0.33, 95 % confidence interval 0.11-0.99) in patients with cirrhosis compared with dipeptidyl peptidase-4 inhibitors (DPP-4is) (Saffo et al., 2021[73]). There is still not enough direct evidence, although some ongoing clinical trials (e.g., NCT05147090 and NCT05254626) are investigating this potential application of SGLT2 inhibitors. There are studies suggesting that SGLT2i therapy is related to reduced levels of liver fibrosis (a main feature of liver cirrhosis) (Taheri et al., 2020[84]). Taheri and coworkers reported that 24 weeks of empagliflozin therapy (10 mg/day) reduced liver stiffness and fibrosis as well as hepatic steatosis in non-diabetic patients with NAFLD (Taheri et al., 2020[84]). A more recent study investigated the long-term impact of SGLT2 inhibitors on patients with T2DM and NAFLD and demonstrated that these drugs diminished fibrosis in the liver tissue (Arai et al., 2022[6]). This trial showed that SGLT2 inhibitors reduce the Fibrosis-4 (FIB-4) index; as a main marker of liver fibrosis; after 48 weeks of treatment in these patients (Arai et al., 2022[6]). Moreover, studies have demonstrated that SGLT2 inhibitions can prevent or reduce fibrotic processes in other tissues *via* different signaling pathways such as oxidative stress, inflammation (e.g. NLRP3 [NOD-, LRR- and pyrin domain-containing protein 3] and MyD88 [Myeloid differentiation primary response 88]), apoptosis, TGF-β1 (transforming growth factor β1)/Smad, and HIF-2α (hypoxia-inducible factor-2α) (Li et al., 2019[54], 2021[55]; Yaribeygi et al., 2019[100]; Quagliariello et al., 2021[68]; Chen et al., 2022[17]; Nan et al., 2022[61]; Yang et al., 2022[94]). Since these pathways are also involved in the pathophysiology of liver cirrhosis (Zhou et al., 2014[108]; Lee et al., 2022[48]; Nan et al., 2022[61]), their modulation using SGLT2 inhibitors in diabetic patients may be a new promising strategy to reduce the risk of liver cirrhosis. However, the results of ongoing trials and mechanistic studies are still required.

### NAFLD 

NAFLD is a common term for a wide range of liver disorders affecting people who drink little to no alcohol and have excess fat accumulation (more than 10 %) around their hepatocytes and liver tissue (Friedman et al., 2018[29]). It is a silent disease with a growing worldwide incidence and is the most common form of chronic liver disorders, affecting as much as about one-quarter of the total population (Sheth and Chopra, 2017[79]; Basu et al., 2022[12]). NAFLD encompasses a range of abnormalities from nonalcoholic fatty liver (NAFL) to nonalcoholic steatohepatitis (NASH) (Cortez-Pinto and Camilo, 2004[22]). NAFL refers to the excess fat stored in the liver, but NASH refers to this condition accompanied by an inflammatory milieu around the hepatocytes (Cortez-Pinto and Camilo, 2004[22]). While obesity and insulin resistance are the major underlying risk factors for NAFL, it is itself a potent inducer of further hepatic complications such as NASH, cirrhosis, acute liver failure, hepatitis, and hepatocellular carcinoma (Bondini and Younossi, 2006[13]; Li et al., 2018[53]; Dhamija et al., 2019[25]). Hence, its management is of high importance to improve liver sufficiency and prevent the aforementioned complications. 

SGLT2 inhibitors have provided some therapeutic benefits for NAFLD (Aso et al., 2019[8]; Chiang et al., 2020[18]; Ala, 2021[3]; Wei et al., 2021[90]). Since fat burning and weight loss are among the outcomes of these drugs, they can effectively reduce the mass of stored fat in the liver and normalize its composition (Shibuya et al., 2018[81]). Meng and colleagues demonstrated that SGLT2 inhibition using empagliflozin attenuates inflammatory responses *via* modulating IL-17/IL-23 and AMPK/mTOR (AMP-activated protein kinase/mammalian target of rapamycin) signaling pathways, and improves liver sufficiency in HFD (high-fat diet) C57BL/6J mice with NAFLD (Meng et al., 2021[58]). They found that empagliflozin reduces NAFLD-induced liver injuries (Meng et al., 2021[58]). A more recent clinical study showed that long-term SGLT2i therapy using empagliflozin provides histological benefits for the liver of patients with T2DM and NAFLD (Akuta et al., 2022[2]). This study found that empagliflozin ameliorates lobular inflammation, steatosis, ballooning, and fibrosis scores in 33 %, 67 %, 0 %, and 33 % of patients, respectively (Akuta et al., 2022[2]). Moreover, a recent meta-analysis of randomized clinical trials has reported that SGLT2i therapy can significantly decline hepatic enzymes and hepatic fat, and improve liver function in T2DM patients with NAFLD (Wei et al., 2021[90]). This study demonstrated that SGLT2 inhibitors decrease liver, visceral, and subcutaneous fat mass and normalize body composition in these patients (Wei et al., 2021[90]). SGLT2 inhibitors can provide liver benefits in patients with NASH (Lai et al., 2020[46]). Lai et al. reported that 24 weeks of empagliflozin therapy provides histological and functional improvements in the liver of T2DM patients with NASH (Lai et al., 2020[46]). They found that empagliflozin attenuates steatosis and ballooning in the liver tissues of these patients (Lai et al., 2020[46]). SGLT2 inhibitors markedly reduce steatosis and normalize the liver tissue composition (Omori et al., 2019[63]) and thus can serve as promising therapeutic agents for patients with NASH. There is still not enough direct clinical evidence, and some ongoing trials are assessing this application of SGLT2 inhibitors (e.g., NCT05254626). However, current knowledge strongly suggests that SGLT2 inhibitors can improve liver sufficiency in patients with NAFLD *via* either promoting fat burning or attenuating the inflammatory processes.

### Hepatocellular carcinoma 

Hepatocellular carcinoma (HCC); the more prevalent form of hepatic cancer in adults; is the leading cause of death in people with cirrhosis and the third most common cause of cancer-dependent deaths worldwide (Balogh et al., 2016[10]). It has been shown that DM and NASH are two main risk factors for HCC development (Fujii et al., 2013[31]; Yu et al., 2013[106]). However, SGLT2i can influence HCC induction or progression, and improve its prognosis (Jojima et al., 2019[42]). Kaji and coworkers demonstrated that SGLT2i therapy using canagliflozin reduces tumor growth by preventing glucose uptake in cultured HuH7 and HepG2 cell lines (Kaji et al., 2018[43]). In an experimental study, canagliflozin provided anti-steatotic and anti-inflammatory effects that in turn decreased NASH development and NASH-to-HCC transition (Jojima et al., 2019[42]). This study demonstrated that canagliflozin induced cell cycle arrest and apoptotic processes, and declined tumor growth in hepatic cells of treated mice (Jojima et al., 2019[42]). In another study, canagliflozin was able to attenuate or delay the onset of NASH and HCC *via* improving lipid profile and fibrotic processes in mice (Shiba et al., 2018[80]). It was reported that canagliflozin decreased the number of tumors in hepatic tissues, declined oxidative stress, and improved glutathione content (Shiba et al., 2018[80]). A more recent meta-analysis of clinical trials showed that SGLT2i therapy is associated with lower mortality in patients with HCC and T2DM (Hendryx et al., 2022[38]). This is consistent with the reported anti-carcinogenic effects of these drugs in other forms of cancer (Wang et al., 2022[89]; Wu et al., 2022[93]). It seems that SGLT2i therapy decreases the risk of HCC and NASH-to-HCC progression. Improvement in lipid profile as well as oxidative, inflammatory, and apoptotic events, and prevention of glucose uptake by SGLT2 inhibitors are possible mechanisms involved in these protective effects (Arvanitakis et al., 2022[7]; Yaribeygi et al., 2022[103]). 

## Conclusion

SGLT2 inhibitors can provide extra-glycemic benefits in the liver tissue (Table 1(Tab. 1); References in Table 1: Aso et al., 2019[8]; Chiang et al., 2020[18]; Coelho et al., 2021[21]; Hendryx et al., 2022[38]; Inoue et al., 2019[40]; Jojima et al., 2019[42]; Leiter et al., 2016[50]; Saffo et al., 2020[72], 2021[73]; Seko et al., 2018[78]; Shiba et al., 2018[80]; Wei et al., 2021[90]). They can normalize LFTs as valuable markers of liver health. Moreover, they can reduce or slow the fibrotic processes and decrease liver tissue stiffness *via* different pathways. Owing to their fat-burning effects, SGLT2 inhibitors reduce the stored lipid droplets in steatotic hepatocytes and normalize liver tissue composition in patients with NAFLD, which in turn prevents subsequent disorders. Finally, these antidiabetics demonstrated potent anti-carcinogenic effects in the liver (and other tissues) and can inhibit NASH to HCC transition or HCC development in the diabetic milieu through several pathways. Taken together, SGLT2 inhibitors are promising glucose-lowering agents with effective hepatoprotective benefits. 

## Notes

Habib Yaribeygi and Amirhossein Sahebkar (Department of Biotechnology, Mashhad University of Medical Sciences, Mashhad, Iran; E-mail: amir_saheb2000@yahoo.com) contributed equally as corresponding author.

## Conflict of interest

The authors declare that they have no conflict of interest in this study. 

## Figures and Tables

**Table 1:**
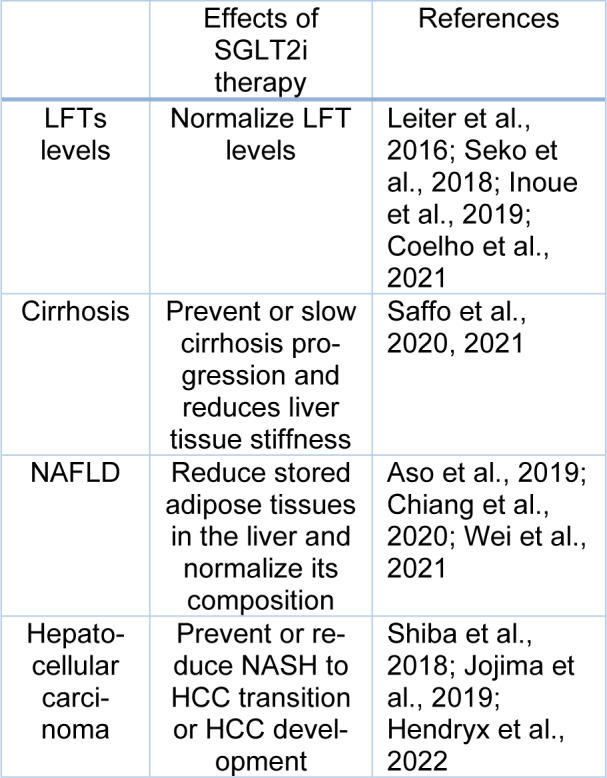
Main interactions between SGLT2 inhibitors and hepatic complications
